# Supplementary material for: Meta-analysis of urinary metabolite GWAS studies identifies novel genome-wide significant loci
Source: Sci Rep. 2025 Jul 1;15:20375. doi: 10.1038/s41598-025-07518-0 (PMC12217396; doi:10.1038/s41598-025-07518-0)
Supplement: Supplementary file 1 — Supplementary Information 1. [file 41598_2025_7518_MOESM1_ESM.docx]

Supplementary Information for:

Meta-Analysis of Urinary Metabolite GWAS Studies Identifies Novel Genome-Wide Significant Loci

Jihan K Zaki^1,2^, Jakub Tomasik^2^, Jade A McCune^1^, Oren A Scherman^1*^, Sabine Bahn^2*^

^1^Department of Chemistry, Lensfield Road, Cambridge, CB2 1EW, Cambridgeshire, United Kingdom

^2^Department of Chemical Engineering and Biotechnology, Philippa Fawcett Drive, Cambridge, CB3 0AS, Cambridgeshire, United Kingdom

* Corresponding authors at: Department of Chemistry, University of Cambridge,

Lensfield Rd, Cambridge CB2 1EW, and Department of Chemical Engineering and

Biotechnology, University of Cambridge, Philippa Fawcett Drive, Cambridge CB3 0AS, UK. E-mail address: sb209@cam.ac.uk (S. Bahn), oas23@cam.ac.uk (O. Scherman). Phone: +44 (0)1223 (3)34151 (S. Bahn), +44 (0)1223 334372 (O. Scherman).

**Supplementary Figures**

**

**Supplementary Figure S1. Linkage disequilibrium plots for all measured SNPs within 1000 kb of the novel lead SNPs for each metabolite.** Points are colored by linkage disequilibrium (r², 1000 Genomes EUR) with the lead SNP (purple, diamond). Gene annotations below the plot are from Ensembl v75 (hg19/GRCh37).


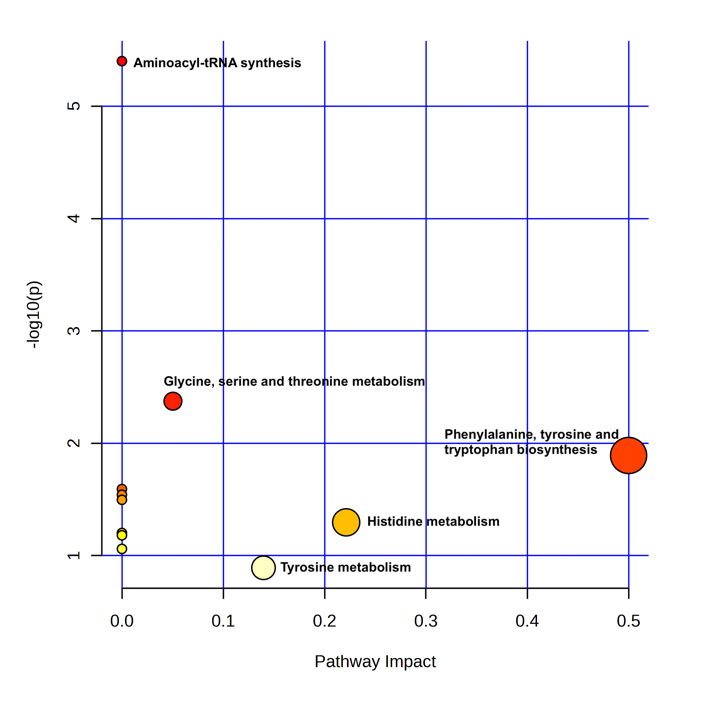

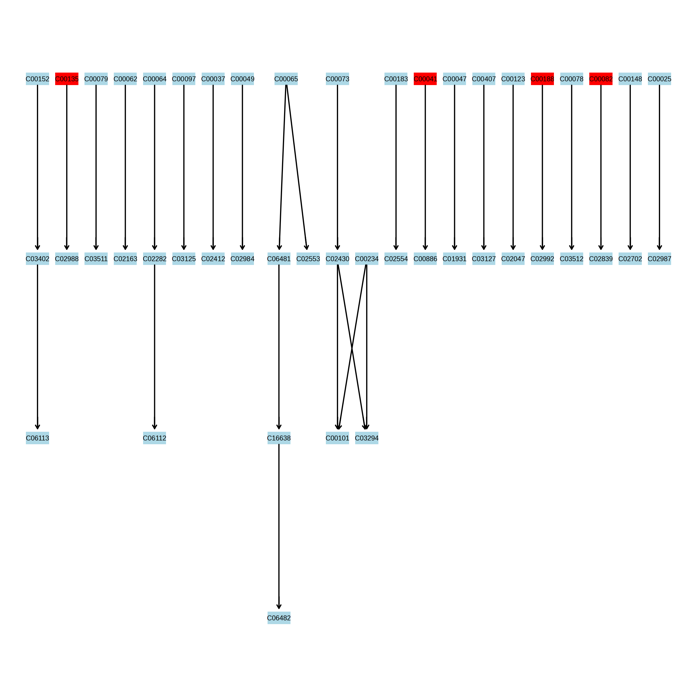


**Supplementary Figure S2. Pathway analysis results for associations near chromosome 17.** All highlighted potential pathways are presented on the left. The most significant pathway, the aminoacyl t-RNA synthesis is presented on the right. Metabolite codes are derived from the Kyoto Encyclopedia of Genes and Genomes with relevant analytes marked in red, and C00188 representing threonine, C00082 representing tyrosine, C00041 representing alanine, and C00135 representing Histidine.
